# Sub-Typing of Rheumatic Diseases Based on a Systems Diagnosis Questionnaire

**DOI:** 10.1371/journal.pone.0024846

**Published:** 2011-09-16

**Authors:** Herman A. van Wietmarschen, Theo H. Reijmers, Anita J. van der Kooij, Jan Schroën, Heng Wei, Thomas Hankemeier, Jacqueline J. Meulman, Jan van der Greef

**Affiliations:** 1 Division of Analytical Biosciences, LACDR, Leiden University, Leiden, The Netherlands; 2 Sino-Dutch Centre for Preventive and Personalized Medicine, Zeist, The Netherlands; 3 Netherlands Metabolomics Centre, Leiden University, Leiden, The Netherlands; 4 Mathematical Institute, Leiden University, Leiden, The Netherlands; 5 Oxrider, Education and Research, Nieuwegein, The Netherlands; 6 TNO, Zeist, The Netherlands; Dana-Farber Cancer Institute, United States of America

## Abstract

**Background:**

The future of personalized medicine depends on advanced diagnostic tools to characterize responders and non-responders to treatment. Systems diagnosis is a new approach which aims to capture a large amount of symptom information from patients to characterize relevant sub-groups.

**Methodology:**

49 patients with a rheumatic disease were characterized using a systems diagnosis questionnaire containing 106 questions based on Chinese and Western medicine symptoms. Categorical principal component analysis (CATPCA) was used to discover differences in symptom patterns between the patients. Two Chinese medicine experts where subsequently asked to rank the Cold and Heat status of all the patients based on the questionnaires. These rankings were used to study the Cold and Heat symptoms used by these practitioners.

**Findings:**

The CATPCA analysis results in three dimensions. The first dimension is a general factor (40.2% explained variance). In the second dimension (12.5% explained variance) ‘anxious’, ‘worrying’, ‘uneasy feeling’ and ‘distressed’ were interpreted as the Internal disease stage, and ‘aggravate in wind’, ‘fear of wind’ and ‘aversion to cold’ as the External disease stage. In the third dimension (10.4% explained variance) ‘panting s’, ‘superficial breathing’, ‘shortness of breath s’, ‘shortness of breath f’ and ‘aversion to cold’ were interpreted as Cold and ‘restless’, ‘nervous’, ‘warm feeling’, ‘dry mouth s’ and ‘thirst’ as Heat related. ‘Aversion to cold’, ‘fear of wind’ and ‘pain aggravates with cold’ are most related to the experts Cold rankings and ‘aversion to heat’, ‘fullness of chest’ and ‘dry mouth’ to the Heat rankings.

**Conclusions:**

This study shows that the presented systems diagnosis questionnaire is able to identify groups of symptoms that are relevant for sub-typing patients with a rheumatic disease.

## Introduction

Pharmacological disease management strategies for rheumatoid arthritis (RA) are for an important part based on trial and error. In general less than 53% of RA patients with a disease duration of less than one year show a positive ACR20 response to treatment [1]. This number goes down to 38% for patients with 5–10 years of RA. Even 30% of RA patients initiating the most effective and expensive treatment option available, anti-TNF therapy, fail to respond [2]. Non-responders are switched to other drugs until one is found that gives the desired effect [3]. A similar trial and error approach is often used for the treatment of osteoarthritis and fibromyalgia as well. The result is that a considerable number of patients experience no benefits from a treatment but just the side effects.

Rheumatoid arthritis patients as well as patients with other rheumatic diseases could benefit substantially from a shift towards a personalized medicine approach which aims to get the right treatment to the right patient, in the right dose, at the right time and via the right route [4], [5]. In the traditional approach, patients are classified as according to criteria specified by the American College of Rheumatology (ACR). A single disease management strategy that is specifically developed for treating the particular type of rheumatic disease will then be applied. A more personalized approach will go beyond the ACR classification and will require much more information about the patient and his or her environment [6]. Specific individual patient situations require specific types of treatment, which can consist of specific drugs, life-style changes, psychological support and other interactions depending on the wish of the patient [7].

The challenge for personalized medicine is to characterize groups of patients and relate these groups to certain treatment options. Modern systems biology technologies such as genomics, proteomics and metabolomics [8]–[10] are currently able to generate an enormous amount of data, which can be seen as signs defined as manifestations that are measured. Several clinical features and molecular markers have been identified for example to sub-type RA patients [11], [12]. Anti-citrullinated protein antibodies positive or negative status is found to be related to distinctive RA risk profiles [13]. More inflamed joints and a higher level of joint destruction was reported in Anti-citrullinated protein antibodies positive RA patients [14]. A large dissimilarity has been found in gene expression profiles of INF-1 high and low sub-types of RA patients, but this is not very clear in the clinical features of patients [15]. Unfortunately, this knowledge has not resulted in personalized health strategies in clinical practice yet, which illustrates that searching for clinically relevant subtypes without clear indications of what to look for and based mostly on signs is difficult.

Symptoms, manifestations that are observed by the patient himself, are on the other hand a subjective type of information which is actually much closer to the phenotype of the patient than signs. Symptoms therefore provide an extra dimension of information. A wide variety of symptoms can be collected related to physical manifestations but also to the psychology, the family, the environment, and worldview of the patient which is well known to play a large role in arthritis [16]–[19].

Diagnosis is the key process in which symptoms and signs are used by a medical practitioner to distinguish the state of a person as different from the ‘normal’ situation. It is used to differentiate one disease from another and it is used to base treatment on. A move towards personalized medicine needs an optimization and refinement of this diagnostic process. One way is to expand it by including more symptoms than currently in use [20].

Chinese medicine diagnosis is a systems diagnosis approach that takes into account a broad spectrum of symptoms (as reported by the patient) as well as signs observed by the practitioner by listening to the body, feeling the body and observation of the patient [21]. Constitutional, behavioral and social aspects are also considered in the diagnosis and the choice of treatment. In Chinese medicine, rheumatoid arthritis as well as other rheumatic diseases fall into a group of diseases termed Bi-syndromes. A Bi-syndrome is characterized by the presence or absence of over 100 symptoms [22].

Symptoms are related to one or more symptom patterns which are called syndromes in Chinese medicine. These patterns or syndromes lead to treatment principles on which particular treatments are based. Recently two particular patterns of symptoms have been studied more closely [23]. These patterns are called Cold and Heat and are general patterns of symptoms much used in Chinese medicine [21], [24]. The Cold pattern can be described as severe pain in a joint or muscle that limits the range of comfortable movement, the pain does not move to other locations. The pain is relieved by applying warmth to the affected area, but increases with exposure to cold. Loose stools are characteristic also, as well as an absence of thirst and clear profuse urine. A thin white tongue coating is seen, combined with a wiry and tight pulse. In contrast, the Heat pattern is characterized by severe pain with hot, red, swollen and inflamed joints. Pain is generally relieved by applying cold to the joints. Other symptoms include fever, thirst, a flushed face, irritability, restlessness, constipation and deep-colored urine. The tongue may be red with a yellow coating and the pulse may be rapid [25].

In a recent study [26] differences have been found between RA Cold and Heat patients when looking at symptoms, gene expression, and metabolomics profiles. Interestingly, the differences turned out to be related to apoptosis, an important biological process. The RA Heat group showed more activity of apoptosis related genes than the RA Cold group. Lu and others found that RA patients with the Cold pattern responded better to biomedical combination therapy (diclofenac, methotrexate, sulfasalazine) than did patients with RA with a Heat pattern, at 12 weeks and 24 weeks of treatment [27]. These findings show that sub-grouping of RA patients using knowledge from Chinese medicine diagnosis can lead to more personalized treatment in RA. Especially the Cold and Heat groups are promising for optimizing treatment.

The objective of the study is to analyze similarities and differences between patients with a rheumatic disease with respect to their symptoms. A questionnaire was therefore designed to establish a systems diagnosis of patients with a rheumatic disease based on a range of symptoms that are used in Chinese and Western medicine. A second objective is to analyze the Cold and Heat status of these patients based on the questionnaire results and an evaluation of the questionnaires by Chinese medicine experts.

Network analysis concepts were used to visualize the relationships between the symptoms and the corresponding syndromes according to Chinese medicine theory, as well as the relationships observed in patients. Categorical principal component analysis was used to find similarities and differences between the patients. The results were interpreted using theoretical and expert knowledge about symptoms and syndromes.

The following part of the analysis focused on the absence and presence of Cold and Heat related symptoms in the patients. Two Chinese medicine experts were asked to rank the Cold and Heat status of each patient on a seven point scale, based on the questionnaire results. These Cold and Heat rankings were introduced as an extra source of information in the categorical principal component analysis. The results were interpreted using Western and Chinese perspectives on arthritis-like diseases. Finally, several suggestions for creating diagnostic tools using the presented systems diagnosis approach will be discussed, which can lead to new opportunities to advance personalized medicine for rheumatic diseases.

## Materials and Methods

### Design of the questionnaire

The questionnaire was designed to establish a systems diagnosis of patients with rheumatic diseases (see Text S1). Symptoms described as related to Bi-syndromes [28] and reviewed by two Chinese medicine experts were used to create a list of 106 questions related to these symptoms divided into nine areas: location of the symptoms, breathing, climate, digestion, emotions & behaviour, quality of the symptoms, changes in the symptoms, pain, and urination. For most of these questions the 7-point Likert scale [29], was used to assess the frequency or the strength of a symptom. A score of 1 means never or not present, while a score of 7 would mean very frequent or very strongly present. Some of the questions were in binary, yes or no, format.

The questionnaire also represents a number of symptoms used in Western medicine to assess disease activity and the response to treatment, for example ‘stiff joints’, ‘joint pain’ and ‘swollen joints’ [30]. In addition to the questions related to symptoms, some general questions were included concerning disease history, medication, and arthritis related blood factors. The full questionnaire is added as supplementary information.

The questionnaire was designed to reflect Chinese thinking and diagnosis by extensive discussions with two Chinese medicine experts. Additionally, the questionnaire was reviewed by the scientific committee of the two Dutch professional organizations for Chinese medicine practitioners in the field of acupuncture (Nederlandse Vereniging voor Acupuntuur) and Nederlandse Artsen Acupunctuur Vereniging).

The medical ethical committee of the Leiden University Medical Center was notified of the study before the questionnaire was send out to patients and waived the need for further approval of the study by the medical ethical committee. All participants were informed about the study the data was going to be used for either by e-mail or verbal communication. All participants gave an informed consent in an e-mail or verbally to this use of the data.

### Study sample

People with one or more rheumatic diseases were invited to participate in this study by completing a questionnaire. A small invitation text was published on the website of the Osteo- and Rheumatoid Arthritis Foundation in Amsterdam, the Netherlands, http://www.reuma-stichting.nl. The invitation was also published in one of the newsletters of the foundation. Additionally, the questionnaire accompanied with an explanation of the purpose of the study was send to all members of the Netherlands Acupuncture Society.

A total of 91 people requested to participate in the study. A questionnaire was send of which 52 were returned. Three questionnaires could not be used for the analysis: one was missing a full page of answers, of another one only the first page was completed and a third questionnaire contained 11 incorrectly filled answers. Table 1 summarizes the types of rheumatic diseases most prevalent in the respondents and the most used medication.

**Table 1:** Characteristics of the respondents.

Rheumatic disease*		Medication	
Osteoarthritis	24	Diclofenac	11
Rheumatoid arthritis	11	Ibuprofen	7
Fibromyalgia	5	Paracetamol	6
Systemic lupus erythematosus	2	Prednison	6
Missing entry	4	Methotrexate	5
Other rheumatic disease	10	Hydroxychloroquine	4
		Tramadol	4
		Etoricoxib	3
		Naproxen	2
		Celecoxib	2
		Other medication	15
		No medication	6
		No entry	5

*10 respondents have a combination of two rheumatic diseases.

### Data screening and recoding

Before data analysis, the consistency of the data was checked. Inconsistent answers to follow-up questions or to questions requiring a severity and frequency score for the same symptom were converted to missing. For example, if the answer to the question ‘Do you feel cold?’ is no, the answer to the follow-up question ‘Is this cold located mostly in the feet or legs?’ should only be no, which is not always the case. The question labeled ‘sighing’ was removed because multiple interpretations were possible and ‘affected parts heavy’ was completely covered by ‘heavy feeling’ and therefore also removed.

Most questions contain categories in which only a few patients had a score, which is unfavorable in CATPCA because it may lead to unstable results [31]. Therefore, we merged categories with a frequency less than 7 (the square root of the number of patients) with an adjacent category. This resulted in 10 variables with only a single category which were therefore removed: ‘vomiting’, ‘nightmares’, ‘joints bend’, ‘joints stretch’, ‘tired with slight exertion’, ‘symptoms wander’, ‘upper part affected’, ‘symptoms appear suddenly’, ‘pain appears slowly’, ‘pain with redness and swelling’. The variable ‘joint pain’ was removed because one of the two categories had only four observations. The final dataset used for the CATPCA analysis contained 93 variables.

### Network analysis

In Chinese medicine the focus is more on relationships between symptoms than on the symptoms themselves. The symptoms involved in the Bi-syndromes form a network of relationships. Network theory [32]–[34] therefore offers a perfect set of concepts to visualize and analyze the network properties of the Bi-syndromes. Cytoscape version 2.6.3 [35], an open source network visualization and annotation package, was used to create a network (graph) of the questionnaire. The position of the nodes was calculated using an algorithm [36] implemented in Cytoscape which minimizes the total length of the edges in the graph. An example is given of how the symptom scores of a patient can be mapped on the network. The questionnaire scores are represented in the graph as the weights of the relationships (edges) between symptoms and the corresponding syndromes. Such a symptom fingerprint can be used by a practitioner to get an overview of the symptom patterns for a particular patient.

### Categorical principal component analysis

To explore the similarities and differences between the patients with respect to the set of 93 variables, Principal Component Analysis (PCA) would be the appropriate method. However, since our data are categorical, standard PCA is not suitable. Nonlinear Principal Component Analysis (NLPCA) is a method that can deal with categorical data and is therefore the method of choice. NLPCA finds the parameters of the PCA model in an iterative process in which “Optimal Scaling” is incorporated. Optimal Scaling is a technique that finds optimal quantifications for categorical variables. The quantifications are optimal in the sense that the percentage of variance of the *quantified* variables accounted for by the principal components is maximal. NLPCA is available in SAS [37] as PRINQUAL [38], [39], and in SPSS [40] as CATPCA [41], [42]. In this study we have used CATPCA in SPSS version 17.0.

The number of principal components was determined using parallel analysis [43], [44] with permuted data [45]. We used 100 data sets with random permutation within all variables. Because CATPCA maximizes the eigenvalues of the first *P* components (with *P* the number of components specified by the user), solutions with different numbers of components are not nested. For instance, the two components in a two-component solution are not equal to the first two components of a three-component solution. Therefore, multiple parallel analyses might be required: If the parallel analysis for the one-component solution shows a significant eigenvalue (greater than the 95^th^ percentile of the random eigenvalues) for the second component, the parallel analysis is repeated for the two-component solution. Then the significance of the third eigenvalue is checked, etc., until the (*P*+1)^th^ eigenvalue of a *P*-component solution is not significant, then *P* is the chosen number of components.

**Figure 1:**
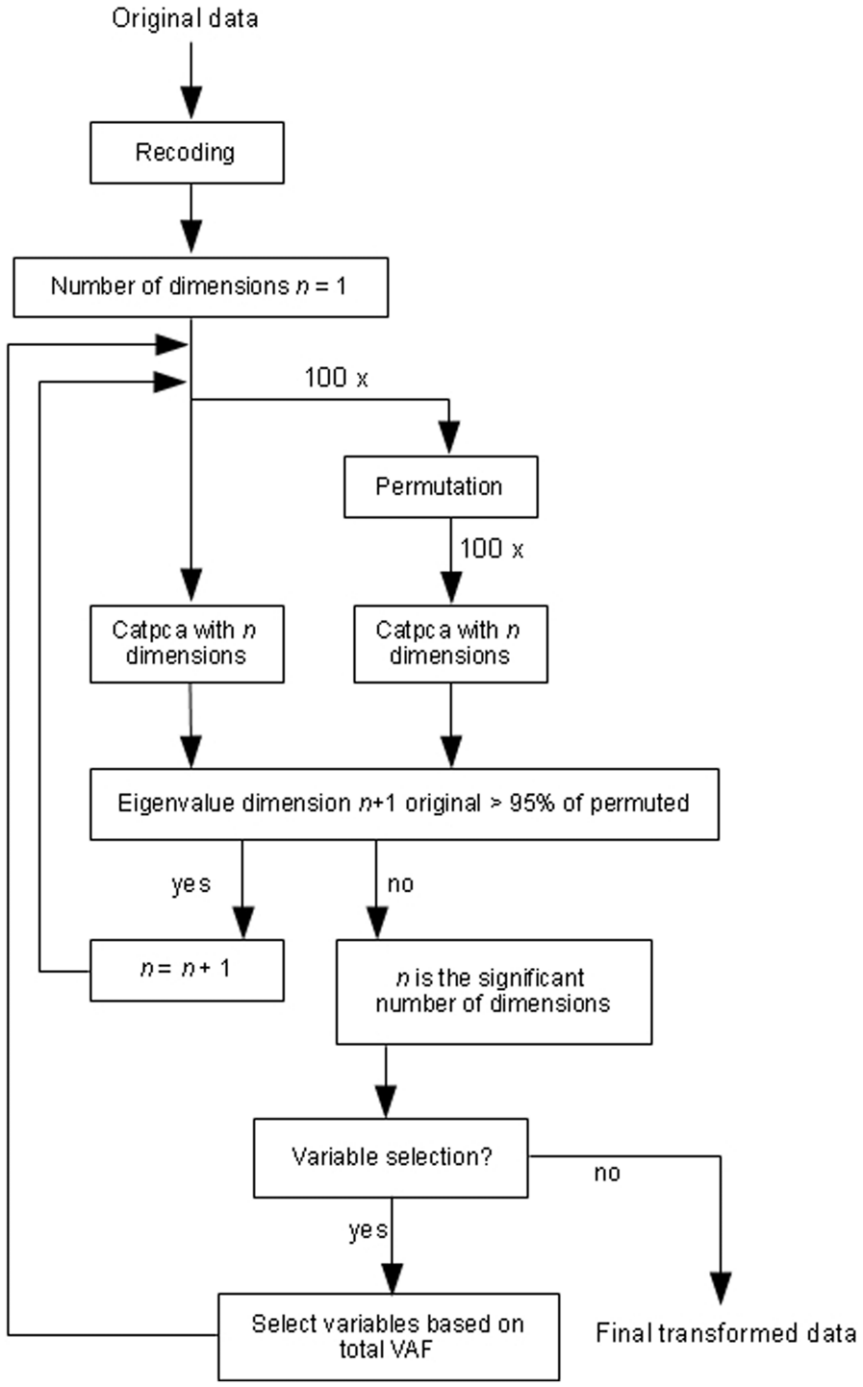
CATPCA data analysis strategy flowchart.

After determining the number of principal components, variables with a total variance accounted for (VAF) ≥50% and another set of variables with a total VAF ≥60% were selected for further analysis. With these two sets of variables the procedure of determining the number of components as described above was repeated (Figure 1).

The loadings resulting from the two CATPCA models were compared with the theoretical relationships between symptoms and syndromes. The two resulting models were interpreted by a Chinese medicine expert. The interpretability determined which of the two sets of variables were the most appropriate for further analysis.

The next part of the analysis focused on the Cold and Heat status of patients and the ability of the questionnaire to provide information on this status. Two Chinese medicine experts (expert 1 and expert 2) were asked which symptoms they deemed most important for Cold and Heat. The two Chinese medicine experts were also asked to determine the Cold and Heat status of each patient based on the questionnaire scores. In the subsequent analysis therefore there are four sources of information: 1) the patient scores on the questionnaire, 2) theoretical Cold and Heat related symptoms, 3) Cold and Heat related symptoms according to experts and 4) Cold and Heat ranking of each patient questionnaire by two experts.

The symptoms that the experts used to rank the Cold and Heat status of the patients were compared with the symptoms connected with Cold and Heat according to theory. Furthermore, to examine the relationship between the symptoms and the Cold and Heat ranking by the experts, these rankings were plotted as ordinally scaled supplementary variables in the CATPCA solution [46]. Supplementary treatment of variables implies that they are projected into the component space, but do not participate in defining the component space. The locations of the Cold and Heat rankings of both experts were then examined respective to each other and the other variables.

To find out which symptoms are most related to the experts Cold and Heat ranking, a semi-supervised analysis was performed. In this analysis the Cold and Heat rankings of the two experts did participate in the model building with a large weight (the ranking variables were included a large number of times in the model) [46]. For this analysis the number of principal components was also determined using the permutation testing approach described above. Due to the large weight of the four ranking variables, they almost completely determine the solution, causing the VAF of the other variables to decrease compared to the unsupervised solution. Questionnaire variables with a total VAF ≥25% in one of both dimensions were selected to build a final model.

## Results and Discussion

### Similarities and differences between patients with a rheumatic disease

**Figure 2:**
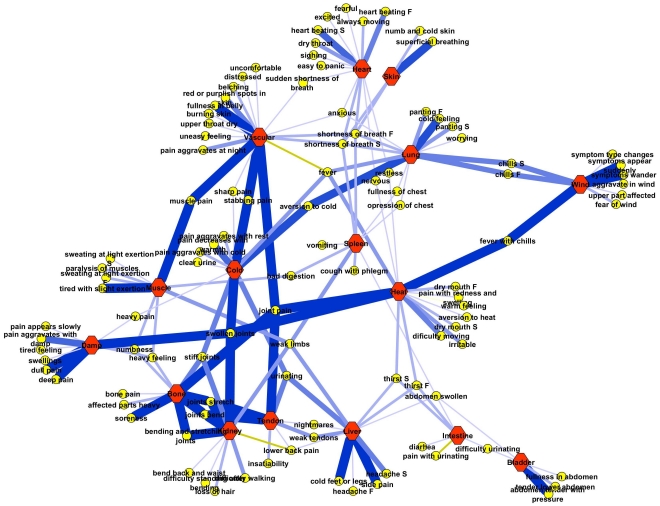
Example of a personalized Bi-syndromes network based on Vangermeersch 1994. The red hexagonal nodes represent the Bi-syndromes (Bi prefix was omitted for brevity), and the yellow circles represent the symptoms and signs related to the Bi-syndromes. The blue lines (edges) represent the relationships between symptoms and syndromes according to theory while the thickness of the lines represent the symptom scores of one patient. Thicker and darker colored edges denote a higher score and thinner, lighter edges denote a lower score.

The theoretical relationships between symptoms used in the systems diagnosis questionnaire and syndromes were visualized as a graph (Figure 2) [20]. The resulting Bi-syndrome network is a bi-partite graph consisting of two types of nodes, the syndromes (red hexagons) and the symptoms (yellow circles). This graph visualizes the relationships between symptoms and syndromes according to Chinese medicine theory. Some symptoms are related to more than one syndrome which might in some instances be referred to as bridge symptoms or bifurcation points. The appearance of such a bridge symptom can indicate a strengthening of the pattern itself, an upcoming change towards another pattern or a complication of the pattern. Many other symptoms are unique for particular syndromes. Certain related syndromes according Chinese medicine theory are positioned close together in the graph. For example prolonged Bone Bi develops into Kidney Bi, two syndromes which are closely related in theory and thus close together in the network. Heart Bi can develop after a long period of Vascular Bi. Intestine and Bladder Bi are two late stages of the disease related to the Hollow Organs [28].

To visually explore the symptom pattern of a patient, the scores on the questionnaire can be mapped on the network. An example of one patient is shown in Figure 2. Edges that are thicker and darker blue denote high scores while light and thin edges represent low scores.

The results of the CATPCA analyses are as follows. Parallel analysis showed four significant principal components for the model containing all the variables. Two sets of variables were selected, one set consisting of 44 variables with a total VAF >50% and another set consisting of 30 variables with a VAF >60% (see Table S1 and Table S2 for the total VAF tables of both sets of variables). Parallel analysis revealed four significant components for the first set of variables and three significant components for the second set. After discussing both models with the Chinese medicine experts the model with fewer variables and components was retained for further analysis.

**Figure 3:**
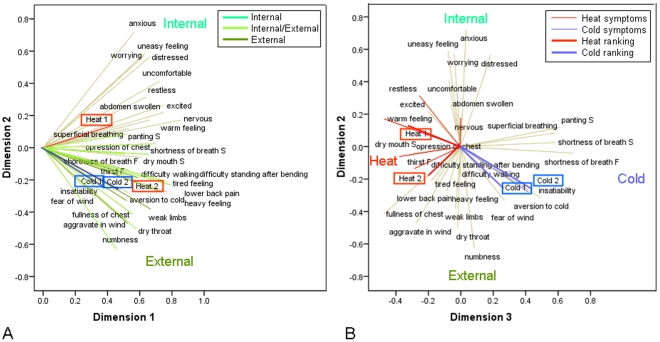
Loading plot of the first two dimensions (panel A) and the second and third dimension (panel B) of the CATPCA model for the 30-variables set. In Panel A, the loading vectors of the symptoms are colored according to their contribution (as indicated by one of the experts) to the Internal or External nature of the disease state. In Panel B, the loading vectors of symptoms which are theoretically related to Cold or Heat are pictured as thin blue or red lines. The supplementary Cold and Heat rankings of the two experts are plotted as thick blue or red lines.

In the component score plots (not shown) no clear groups of patients could be observed. The location of the objects in the score plots was compared with the type of arthritis the patients suffered from. No grouping of patients with a similar type of arthritis could be found. The loadings of the first two components of the CATPCA model are shown in Figure 3A. The length of a loading indicates the amount of variance explained by that loading and the distance between two loadings indicates their similarity. All the loadings are positive in the first dimension (40.2% explained variance) indicating a general factor for which no clear interpretation was found. When the loadings in the second dimension (12.5% explained variance) are compared with the theoretical relationships between symptoms and the corresponding syndromes, two groups of loadings stand out. Five variables are theoretically related to the external pathogens Wind (‘aggravate in wind’, ‘fear of wind’), Cold (‘aversion to cold’) and Heat (‘thirst’, ‘dry mouth s’). Three of these symptoms, ‘aggravate in wind’, ‘fear of wind’ and ‘aversion to cold’, have a fairly high negative loading in the second dimension. Nine variables are related to Chinese Organ concepts such as Heart (‘anxious’, ‘restless’, ‘excited’, ‘nervous’), Vascular (‘anxious’, ‘uncomfortable’, ‘distressed’, ‘uneasy feeling’) and Lung (‘panting s’, ‘worrying’). Four of these symptoms, ‘anxious’, ‘worrying’, ‘uneasy feeling’ and ‘distressed’, have a fairly high positive loading in the second dimension. Two symptoms which are related to Damp, ‘heavy feeling’ and ‘tired feeling’, have a very low loading in the second dimension.

Looking at the distribution of symptoms from a Western medicine perspective reveals that the most common symptoms for rheumatoid arthritis (RA) ‘joint pain’, ‘swollen joints’ and ‘stiff joints’ do not contribute to the variation between the patients. On the other hand two other RA symptoms ‘tired feeling’ and ‘weak limbs’ are present but close together. The main osteoarthritis symptoms ‘pain’ and ‘stiffness’ are not present. Fibromyalgia is characterized by pain and stiffness as well, but also by ‘anxious’ and ‘distressed’, which have a high loading in the second dimension.

According to the Chinese medicine experts the second dimension is related to the Internal or External stage of the disease, one of the key diagnostic concepts used in Chinese medicine [21]. In the Internal stage the organs are affected while in the External stage the skin, muscles and channels are affected. The reaction of the body to the external pathogens Wind, Cold, and Damp that cause the arthritis is expressed by the symptoms that can be observed in the lower right part of Figure 3A. This indicates the first, external stage of the disease when the body defends itself against the invasion of the external pathogens. The appearance of Heat (‘dry mouth s’ and ‘thirst’) and Damp (‘heavy feeling’ and ‘tired feeling’) symptoms, indicates a transformation of Cold into Heat via Damp. Patients in this stage of the disease will have a low score in the second dimension. A high positive loading in the second dimension indicates a more chronic stage of the disease, in which patients will present Organ symptoms. If the position of the objects in the component score plots is compared to the loadings, it is possible to get an indication of the stage of the disease according to Chinese medicine theory for each patient. The Internal versus External interpretation of the second dimension is in agreement with the distribution of the symptoms that are in theory related to the External or Internal stage of the disease as marked with different colors in Figure 3.

### The Cold and Heat status of rheumatic patients

**Figure 4:**
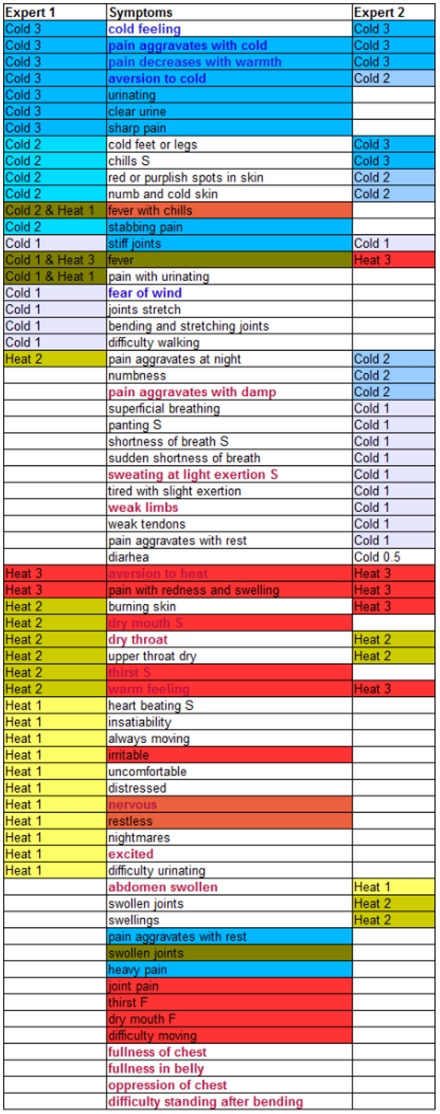
Cold and Heat related symptoms. In the first and third column the symptoms used by the experts for the Cold (blue) and Heat (red) ranking are marked. The importance is shown as a number behind the Cold and Heat label and by the color, dark blue or red is more important. The second column shows the names of the symptoms. Symptoms with a blue background are related to Cold according to theory, the red background ones are related to Heat according to theory. Symptoms with a brown background are related to both Cold and Heat. The table also shows which symptoms are important for the Cold and Heat ranking according to the forced classification model in bold type face and in red or blue.

Figure 3B shows the second and third dimension of the CATPCA model. While the second dimension is mostly related to the Internal and External stage of the disease, the third dimension (10.4% explained variance) is related to the Cold or Heat status of the patients. Symptoms related to Heat and Cold according to theory are colored red and blue respectively in Figure 3 and are indicated in Figure 4. Five symptoms that are theoretically related to Heat have a negative loading in the third dimension (‘restless’, ‘nervous’, ‘warm feeling’, ‘dry mouth s’ and ‘thirst’). The group of symptoms with the highest positive loadings in the third dimension (‘panting s’, ‘superficial breathing’, ‘shortness of breath s’ and ‘shortness of breath f’) are related to Qi deficiency. One symptom (‘aversion to cold’) theoretically related to Cold also has a fairly high positive loading in the third dimension.

The Cold and Heat rankings of the experts, plotted as supplementary variables in Figure 3, have fairly high loadings in the third dimension indicating that this dimension is related to the Cold and Heat rankings. The Cold rankings are related to the other high positive loadings in the third dimension (‘panting s’, ‘superficial breathing’, ‘shortness of breath s’ and ‘shortness of breath f’). One expert indicated to have used these symptoms to rank the Cold status of the patients (Figure 4). The Cold rankings are much closer together than the Heat rankings indicating that the two experts agree more on the Cold status of the patients than on the Heat status.

The experts were asked to indicate which symptoms they used for the ranking of the Cold and Heat status of the patients. In Figure 4 the symptoms reported by the two experts are given. Additionally, the symptoms theoretically related to Cold and Heat are indicated by the red and blue color in the center column. In the figure the overlap in symptom use between theory and the experts is visualized, as well as the overlap between the two experts. The Cold symptom ‘aversion to cold’ with a large loading in dimension three shown in Figure 3B was indeed indicated by both experts as important, although one expert assigned this symptom a lower status. Of the Heat symptoms with a large loading in dimension three ‘warm feeling’ was deemed important by both experts. ‘Dry mouth s’, ‘nervous’ and ‘restless’ were important for one expert, while ‘thirst f’ was mentioned by neither expert. Figure 4 also shows that both experts used a larger set of symptoms to rank Cold and Heat than indicated by theory. Furthermore, both experts indicated to have used symptoms that are not related to Cold and Heat according to theory. This might be due to differences between various Chinese medicine schools and to experience with using symptoms in daily practice.

**Figure 5:**
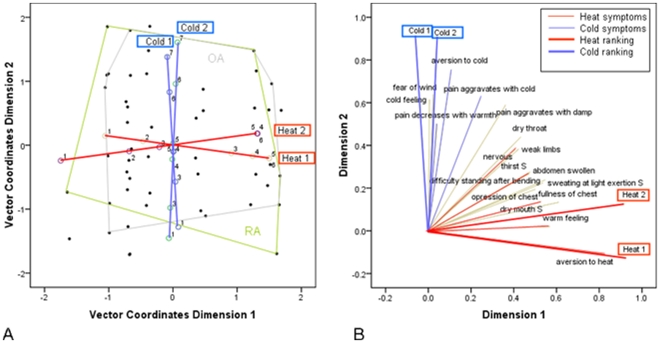
Panel A shows a bi-plot based on a semi-supervised CATPCA model including 19 variables and the 4 expert ranking variables. The four expert rankings are shown on which the categories after transformation are marked by circles. The outline of the group of rheumatoid arthritis (RA) patients is shown as well as the outline of the group of osteoarthritis patients (OA). In Panel B the loadings are shown. The loadings corresponding to symptoms which are related to Cold or Heat according to theory are represented by thin blue or red lines respectively. The Cold and Heat expert rankings are the thick blue en red lines respectively.

In the following analysis the Cold and Heat rankings are introduced into the model with a large weight to find the symptoms that are most closely related to the expert rankings, based on the patients scores. Figure 5A is a bi-plot in which the patients scores and Cold and Heat ranking loadings are both plotted in the component space. The position of a patient point relative to the Cold and Heat loadings indicates the Cold and Heat ranking of the patient. Patients in the upper part of the figure for example have a high Cold ranking while patients in the right part of the figure have a high Heat ranking. Figure 5A shows that the distances between the various ranking categories is not equal. Additionally the distance between category 3 in Heat 1 and Heat 2 is large indicating that a Heat ranking of 3 for expert 1 should be interpreted very different from a Heat ranking of 3 for expert 2. To see whether the Cold or Heat ranking has any relationship with the type of arthritis the patients suffer from the outline of the largest groups of patients, the rheumatoid arthritis (RA) and osteoarthritis (OA) patients, are marked by lines. Clearly, the scores of the OA and RA patients are overlapping and is therefore unrelated to the Cold and Heat rankings.

In Figure 5B shows the loadings resulting from the forced classification analysis. The Heat rankings have a high positive loading in the first dimension and the Cold rankings have a high positive loading in the second dimension. This loadings plot reveals which symptoms, and related questions, are most related to the Cold and Heat rankings. ‘Aversion to cold’, ‘fear of wind’ and ‘pain aggravates with cold’ are most related to the Cold rankings and ‘aversion to heat’, ‘fullness of chest’ and ‘dry mouth’ to the Heat rankings. ‘Aversion to cold’ and ‘fear of wind’ also have a fairly high positive loading in the third dimension (indicating Cold) of the unsupervised approach (Figure 3B). ‘Fullness of chest’ and ‘dry mouth’ have a fairly high negative loading in the third dimension (indicating Heat) of the unsupervised approach.

The results of this study are summarized in Figure 4. First of all the symptoms used in theory to determine the Cold and Heat status of patients are indicated with a blue and red background respectively. ‘Fever’ and ‘swollen joints’ have a brown background since they are bridge symptoms between Heat and Cold. Secondly, the figure shows that both experts reported they used most of the theoretical symptoms. Seven symptoms that were not used by either expert are placed at the bottom of the list. Thirdly, the figure shows the agreement and disagreement between the experts on symptom use. In the fourth place, the symptoms resulting from the forced classification analysis are marked by bold type face.

Of the Western symptoms for rheumatoid arthritis ‘joint pain’ is indicated as a Heat symptom in Chinese diagnosis, ‘stiff joints’ is indicated as a Cold symptom and ‘swollen joints’ is indicating both Cold and Heat. However neither expert used ‘swollen joints’ and ‘joint pain’ to rank the Cold or Heat status of the patients. ‘Weak limbs’, another RA symptom showed up in the forced classification as an important indicator for Heat. Some pain related symptoms which can be present in various rheumatic diseases appear to be relevant for Cold and Heat ranking. ‘Pain with redness and swelling’ is indicated by both experts and theory as an important Heat symptom. ‘Stabbing pain’ on the other hand is indicated by one expert as a Cold symptom. ‘Heavy pain’ was not mentioned by the experts, but according to theory it is a Cold symptom. Additionally the results of the analysis show that emotional symptoms more prevalent in fibromyalgia patients are more related to Heat, especially the symptom ‘nervous’ is mentioned in theory as a Heat symptom and is also an important Heat indicator in the forced classification results.

The combination of the patient questionnaire scores information with the analysis and expert rankings resulted in a set of symptoms that are most qualified to develop into a tool to determine the Cold and Heat status of patients in a clinical setting.

### Conclusions

This study introduced a systems diagnosis approach, the collection of a large number of symptoms that are usually not used in clinical diagnosis in Western medicine, as an additional dimension of looking at patients with a rheumatic disease. Individual patient scores on this questionnaire can be visually presented in a graph to help the interpretation of the relationships between the symptoms occurring in that patient (Figure 2). This new method of ‘symptom fingerprinting’ is comparable to other systems biology fingerprinting tools to determine disease state, risk for a disease, and chances of treatment effect [47]–[49].

The systems diagnosis questionnaire results of 49 patients with a rheumatic disease in this study reveal two interesting and significant dimensions of information. One dimension is related to the stage of the disease with the key symptoms ‘anxious’, ‘worrying’, ‘uneasy feeling’ and ‘distressed’ for the Internal stage, and ‘aggravate in wind’, ‘fear of wind’ and ‘aversion to cold’ for the External stage. The concept of Internal and External is widely used in Chinese medicine to choose the right treatment option. The fact that this concept explains 12.5% of the total variation in the data shows that it might be a relevant difference in other patients with rheumatic diseases as well.

The second interesting dimension is related to the Cold and Heat status of the patients, explaining 10.4% of the variation in the data. The key symptoms are ‘panting s’, ‘superficial breathing’, ‘shortness of breath s’, ‘shortness of breath f’ and ‘aversion to cold’ for the Cold status and ‘restless’, ‘nervous’, ‘warm feeling’, ‘dry mouth s’ and ‘thirst’ for the Heat status. A forced classification approach revealed that ‘Aversion to cold’, ‘fear of wind’ and ‘pain aggravates with cold’ are related to the Cold rankings and ‘aversion to heat’, ‘fullness of chest’ and ‘dry mouth’ are related to the Heat rankings by two Chinese medicine experts in these 49 patients. The characterization of the Cold and Heat status in this study is limited by the patient reported symptoms and might be improved by including observations of an expert of the patient.

We believe that the future of medicine lies in an integration of perspectives on disease, health, prevention and medicine. Looking in more detail and at the same time more comprehensively at patients is the way forward towards personalized medicine [5], [50]. One step in that direction is finding new sub-groups of patients and optimizing treatment for these sub-groups. For Chinese medicine practitioners it is standard to take the Internal or External status as well as the Cold or Heat status into account for choosing the best treatment option. This study characterizes the symptoms related to these subgroups which might be used to develop a diagnostic tool to diagnose these subgroups in clinical practice. Further studies are needed to examine differences in response to medication by Cold and Heat sub-groups of arthritis patients. In this way the combination of Chinese medicine concepts with Western therapeutic options offers exciting opportunities for more personalized treatment and eventually personalized health.

## Supporting Information

Text S1 Systems diagnosis questionnaire (in Dutch).
(DOC)

Table S1 VAF table for the analysis presented in Figure 3.
(DOC)

Table S2 VAF table for the analysis presented in Figure 5.
(DOC)
