# Supplementary material for: Sub-Typing of Rheumatic Diseases Based on a Systems Diagnosis Questionnaire
Source: PLoS One. 2011 Sep 16;6(9):e24846. doi: 10.1371/journal.pone.0024846 (PMC3174973; doi:10.1371/journal.pone.0024846)
Supplement: Text S1 — Systems diagnosis questionnaire (in Dutch). (DOC) [file pone.0024846.s001.doc]

**Onderzoek**
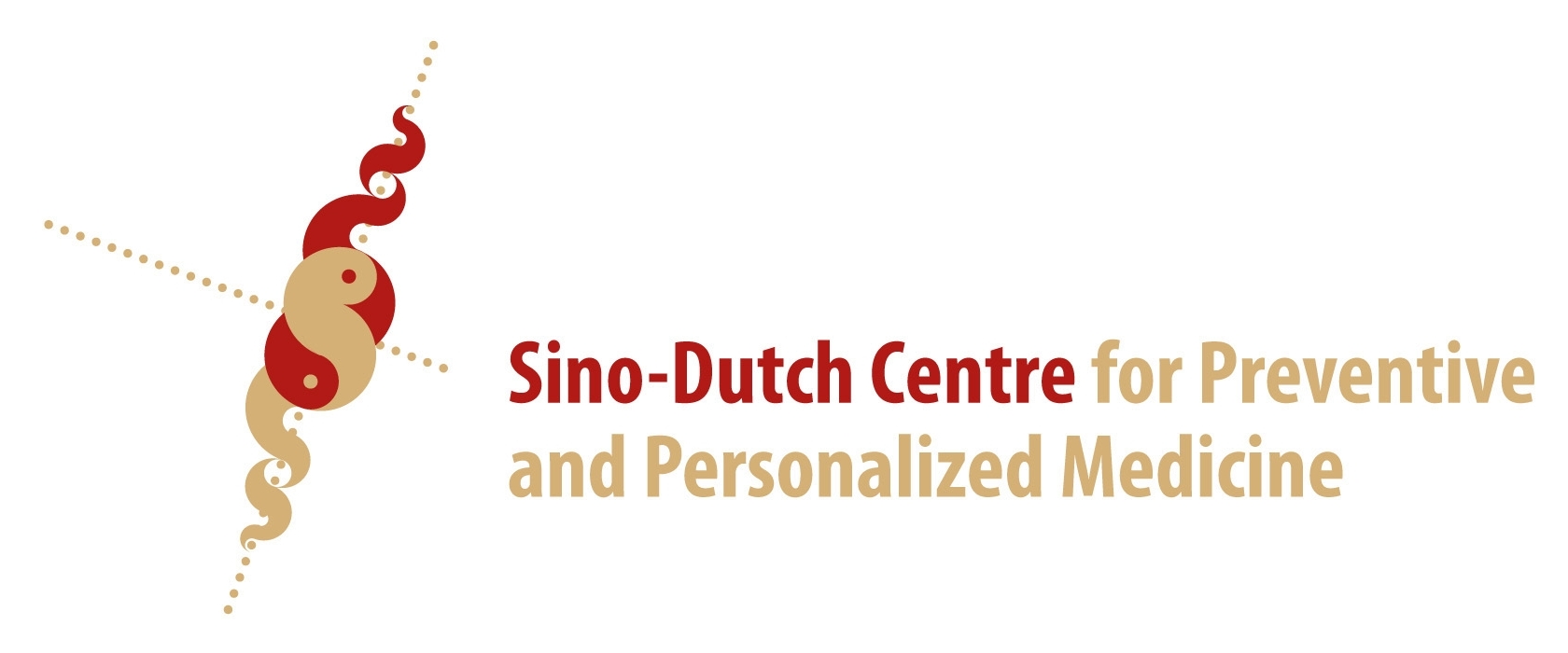
 **diagnose van patiënten met een reumatische aandoening**

Hartelijk dank voor het meewerken aan dit onderzoek naar de diagnose van patiënten met een reumatische aandoening. Het invullen van de vragen zal ongeveer een kwartier duren.

Gelieve alle vragen in te vullen. Indien geen antwoord mogelijk is dit graag vermelden. Alle informatie zal vertrouwelijk worden behandeld.

Voor het beantwoorden van de vragen wordt regelmatig gevraagd naar de Mate en of Frequentie van een symptoom. Hiertoe dient u een waarde van 1 tot en met 7 in te vullen, waarbij 1 overeenkomt met nooit of niet, en waarbij 7 overeenkomt met zeer ernstig of zeer vaak.

Succes!

| **Datum** |  |
| --- | --- |

| **1. Algemene informatie** | | | |
| --- | --- | --- | --- |
| Leeftijd aanvang ziekte |  | C-reactive protein |  |
| Reumafactor |  | Hb |  |
| ESR |  | ACR klasse |  |

| Reguliere medische diagnose | | |
| --- | --- | --- |
|  | | |
| Wie heeft de diagnose gesteld? | | |
|  | | |
| Reguliere medicatie + bijwerkingen | | |
|  | | |
| Overige medicatie | | |
|  | | |
| Korte ziekte geschiedenis | | |
|  | | |
| **2. Locatie van de symptomen** | | **Antwoord** |
| Ervaart U een vol gevoel in de borst?  - is dit gevoel vooral gelocaliseerd in het midden boven deel van de buik? | | Mate (1-7): [ ]  Ja/Nee |
| Ervaart U een druk op de borst? | | Mate (1-7): [ ] |
| Ervaart U hoofdpijnen? | | Mate (1-7): [ ]  Frequentie (1-7): [ ] |
| Ervaart U een abnormaal bewustzijn van het kloppen van uw hart, stoort dit andere gedachten? | | Mate (1-7): [ ]  Frequentie (1-7): [ ] |
| Ervaart U een brandend gevoel van de huid? | | Mate (1-7): [ ] |
| Heeft U haaruitval? | | Mate (1-7): [ ] |
| Heeft U rode of paarsachtige plekken in of onder de huid? | | Mate (1-7): [ ] |
|  | |  |
| **3. Ademhaling** | | **Antwoord** |
| Is uw ademhaling oppervlakkig? | | Mate (1-7): [ ] |
| Heeft U last van hijgen? | | Mate (1-7): [ ]  Frequentie (1-7): [ ] |
| Ervaart U kortadmenigheid?  - Vindt dit plotseling plaats? | | Mate (1-7): [ ]  Frequentie (1-7): [ ]  Ja/Nee |
| Ervaart U een droge mond? | | Mate (1-7): [ ]  Frequentie (1-7): [ ] |
| Ervaart U regelmatig een droge keel?  - Is deze droogheid gelocaliseerd in het bovenste deel van de keel? | | Frequentie (1-7): [ ]  Ja/Nee |
| Geeft U slijm op bij het hoesten? | | Frequentie (1-7): [ ] |
|  | |  |
| **4. Klimaat** | | **Antwoord** |
| Voelt U zich koud?  - Is deze koude vooral in de voeten of benen aanwezig? | | Mate (1-7): [ ]  Ja/Nee |
| Ervaart U dorst? | | Mate (1-7): [ ]  Frequentie (1-7): [ ] |
| Ervaart U rillingen? | | Mate (1-7): [ ]  Frequentie (1-7): [ ] |
| Voelt U zich warm? | | Mate (1-7): [ ] |
| Heeft U soms koorts?  - Gaat de koorts soms gepaard met rillingen? | | Frequentie (1-7): [ ]  Ja/Nee |
| Ervaart U moeilijkheden met bewegen? | | Mate (1-7): [ ] |
| Heeft U afkeer van hitte? | | Mate (1-7): [ ] |
| Heeft U afkeer van koude? | | Mate (1-7): [ ] |
| Ervaart U spontaan zweten of zweten bij de geringste inspanning? | | Mate (1-7): [ ]  Frequentie (1-7): [ ] |
|  | |  |
| **5. Spijsvertering** | | **Antwoord** |
| Is uw onderbuik pijnlijk of erg gevoelig?  - Wordt dit gevoel versterkt door er op te duwen? | | Mate (1-7): [ ]  Ja/Nee |
| Voelt uw buik gezwollen of uitgezet aan? | | Mate (1-7): [ ] |
| Ervaart U een vol gevoel in de buik? | | Mate (1-7): [ ] |
| Heeft U last van diarree? | | Frequentie (1-7): [ ] |
| Hoe vaak moet U boeren laten? | | Frequentie (1-7): [ ] |
| Hoe vaak moet U overgeven? | | Frequentie (1-7): [ ] |
| Ervaart U een onverzadigbare drang om te eten? | | Frequentie (1-7): [ ] |
| Ervaart U een slechte vertering? | | Mate (1-7): [ ] |
|  | |  |
| **6. Emoties & Gedrag** | | **Antwoord** |
| Bent U altijd in beweging? | | Mate (1-7): [ ] |
| Bent U gemakkelijk geïrriteerd? | | Frequentie (1-7): [ ] |
| Voelt U zich ongemakkelijk? | | Frequentie (1-7): [ ] |
| Vreest U wind of tocht? | | Mate (1-7): [ ] |
| Bent U bang? | | Mate (1-7): [ ] |
| Raakt U gemakkelijk in paniek? | | Frequentie (1-7): [ ] |
| Voelt U zich gestrest? | | Frequentie (1-7): [ ] |
| Maakt U zich zorgen? | | Frequentie (1-7): [ ] |
| Heeft U een ongemakkelijk gevoel? | | Frequentie (1-7): [ ] |
| Piekert U veel? | | Frequentie (1-7): [ ] |
| Voelt U zich nerveus? | | Frequentie (1-7): [ ] |
| Voelt U zich rusteloos? | | Frequentie (1-7): [ ] |
| Ervaart U nachtmerries? | | Frequentie (1-7): [ ] |
| Voelt U zich opgewonden? | | Mate (1-7): [ ] |
| Moet U vaak zuchten? | | Frequentie (1-7): [ ] |
|  | |  |
| **7. Kwaliteit van de symptomen** | | **Antwoord** |
| Kunt U de aangedane gewrichten buigen?  - Kunt U de aangedane gewrichten strekken? | | Ja/Nee  Ja/Nee |
| Is het buigen en strekken van uw gewrichten beperkt? | | Mate (1-7): [ ] |
| Ervaart U een zwaar gevoel in uw ledematen, lichaam en of hoofd? | | Mate (1-7): [ ] |
| Voelen de aangedane delen van uw lichaam zwaar aan? | | Mate (1-7): [ ] |
| Ervaart U een moe gevoel in uw ledematen, lichaam en of hoofd? | | Mate (1-7): [ ] |
| Ervaart U gevoelloosheid in het lichaam en ledematen? | | Mate (1-7): [ ] |
| Voelt uw huid gevoelloos en koud aan? | | Mate (1-7): [ ] |
| Voelt U stijfheid in uw gewrichten? | | Mate (1-7): [ ] |
| Heeft U gezwollen gewrichten? | | Mate (1-7): [ ] |
| Heeft U zwellingen in uw lichaam? | | Mate (1-7): [ ] |
| Voelt U zich moe na de geringste inspanning? | | Ja/Nee |
| Voelen uw vier ledematen zwak? | | Mate (1-7): [ ] |
| Voelen uw pezen zwak? | | Mate (1-7): [ ] |
| Ervaart U moeilijkheden met het lopen? | | Mate (1-7): [ ] |
| Heeft U een gebogen rug en middel?  - Heeft U moeite met recht op staan na buigen? | | Mate (1-7): [ ]  Mate (1-7): [ ] |
|  | |  |
| **8. Veranderingen in de symptomen** | | **Antwoord** |
| Verplaatsen de symptomen zich? | | Ja/Nee |
| Worden de symptomen erger wanneer het buiten winderig is? | | Mate (1-7): [ ] |
| Is het bovenste deel van uw lichaam gemakkelijker aangedaan dan het onderste deel van uw lichaam? | | Ja/Nee |
| Verschijnen de symptomen plotseling? | | Ja/Nee |
| Verandert het type symptomen? | | Mate (1-7): [ ] |
|  | |  |
| **9. Pijn** | | **Antwoord** |
| Heeft U last van lage rugpijn? | | Mate (1-7): [ ] |
| Heeft U pijn? | | Mate (1-7): [ ] |
| Waar ervaart U pijn? | [Spieren] [gewrichten] [botten] [onderrug] [zij] | |
| Wat voor soort pijn ervaart U? | [Zeurend] [stekend] [scherp] [diep] [zwaar] [dof] | |
| Komt de pijn langzaam, gradueel op? | | Ja/Nee |
| Ervaart U verlamming in uw spieren? | | Mate (1-7): [ ] |
| Wordt de pijn erger met vochtig, mistig weer? | | Mate (1-7): [ ] |
| Wordt de pijn 's nachts erger? | | Mate (1-7): [ ] |
| Wordt de pijn erger bij koude en koud weer? | | Mate (1-7): [ ] |
| Wordt de pijn erger door te rusten? | | Mate (1-7): [ ] |
| Gaat de pijn gepaard met roodheid en zwelling? | | Ja/Nee |
| Wordt de pijn minder door warmte en beweging? | | Mate (1-7): [ ] |
|  | |  |
| **10. Urineren** | | **Antwoord** |
| Hoe vaak moet U plassen? | | Frequentie (1-7): [ ] |
| Is de urine helder van kleur? | | Mate (1-7): [ ] |
| Ervaart U moeilijkheden bij het plassen? | | Mate (1-7): [ ] |
| Ervaart U pijn bij het plassen? | | Mate (1-7): [ ] |

Hierbij verklaar ik deze vragenlijst naar waarheid te hebben ingevuld.

| Handtekening patiënt: |
| --- |

Eventueel kunt u hieronder uw adresgegevens opgeven om op de hoogte gehouden te worden van het onderzoek.

| Naam |  |
| --- | --- |
| Adresgegevens |  |
| E-mail |  |

Hartelijk dank voor uw medewerking!

*Drs. Herman van Wietmarschen*

*Leiden Universiteit, LACDR, ABS, Einsteinstraat 55, 2333 CC, Leiden, reumavragenlijst@gmail.com*
