# Supplementary material for: Sub-Typing of Rheumatic Diseases Based on a Systems Diagnosis Questionnaire
Source: PLoS One. 2011 Sep 16;6(9):e24846. doi: 10.1371/journal.pone.0024846 (PMC3174973; doi:10.1371/journal.pone.0024846)
Supplement: Table S2 — VAF table for the analysis presented in Figure 5. (DOC) [file pone.0024846.s003.doc]

Table S3: VAF table CATPCA analysis Figure 5.
